# A historical journey through total knee arthroplasty: the evolutionary milestones

**DOI:** 10.1007/s00264-026-06976-z

**Published:** 2026-08-07

**Authors:** Julien Wegrzyn, Mo Saffarini

**Affiliations:** 1 Department of Orthopedic Surgery and Trauma, University Hospital of Lausanne and University of Lausanne, Lausanne, Switzerland; 2 ReSurg, Nyon, Switzerland

**Keywords:** Total knee arthroplasty, History, Implant design

## Abstract

**Purpose:**

This study aims to examine historical progressions to demonstrate how current debates in alignment philosophy, implant personalization, robotic surgery, and ligament management serve as direct extensions of past concepts.

**Methods:**

The history of TKA was divided into four major evolutionary phases: the era of experimentation between 1860 and 1970, the period of implant design and biomechanical standardization between 1970 and 2000, the rise of personalization and morphologic understanding between 1985 and 2020, and the modern era of perioperative and technological evolution beginning in the early 2000’s.

**Results:**

One of the earliest total joint replacement concepts was introduced in 1890 by Gluck, who implanted an ivory hinged knee prosthesis fixed with primitive cement materials. Modern hinged knee arthroplasty emerged during the 1950s to address severe arthritis and ligamentous instability, which constrained out-of-plane movement by allowing only flexion–extension. During the 1980s, modularity and mobile-bearing concepts gained widespread popularity. Metal-backed tibial components allowed polyethylene exchange and improved intraoperative flexibility, a concept exemplified by the Insall Burstein Posterior Stabilized II prosthesis. Beginning in the late 1980s and accelerating during the 1990s and early 2000s, researchers increasingly recognized that standardized implant geometries did not accurately reproduce native anatomy in many patients. During the 2000s and 2010s, alignment philosophy underwent a major paradigm shift. The twenty-first century introduced major advances in digital surgery.

**Conclusion:**

The future of total knee arthroplasty will be shaped by advances in implant design, surgical methods, digital technologies, and biologic reconstruction. These trends continue the goal of mimicking native knee anatomy, biomechanics, and function. Future implants will likely shift from standard components to more personalized solutions.

## Introduction

Total knee arthroplasty (TKA) is considered one of the most successful orthopaedic procedures in modern medicine, with survival rates exceeding 90% at 15 to 20 years in many modern series [1]. The evolution of TKA reflects more than a century of continuous innovation, driven by advances in anatomy, biomechanics, biomaterials, radiology, industrial manufacturing, implant design, fixation techniques, and evolving surgical philosophy. Early attempts at knee reconstruction were largely experimental and often associated with poor outcomes; however, these pioneering procedures established the conceptual foundations of modern arthroplasty.

Total knee arthroplasty has transformed the management of end-stage knee arthritis by reliably relieving pain and restoring function. Despite its current success, the modern knee prosthesis is the result of a long and complex historical evolution extending over more than 150 years. Unlike hip arthroplasty, the development of TKA was particularly challenging because the knee is not a simple hinge joint. Its motion depends on the coordinated interaction of articular geometry, ligament balance, muscle forces, limb alignment, femoral rollback, patellofemoral tracking, and rotational alignment. Consequently, many early prosthetic concepts failed because they did not adequately reproduce native knee kinematics.

**Fig. 1:**
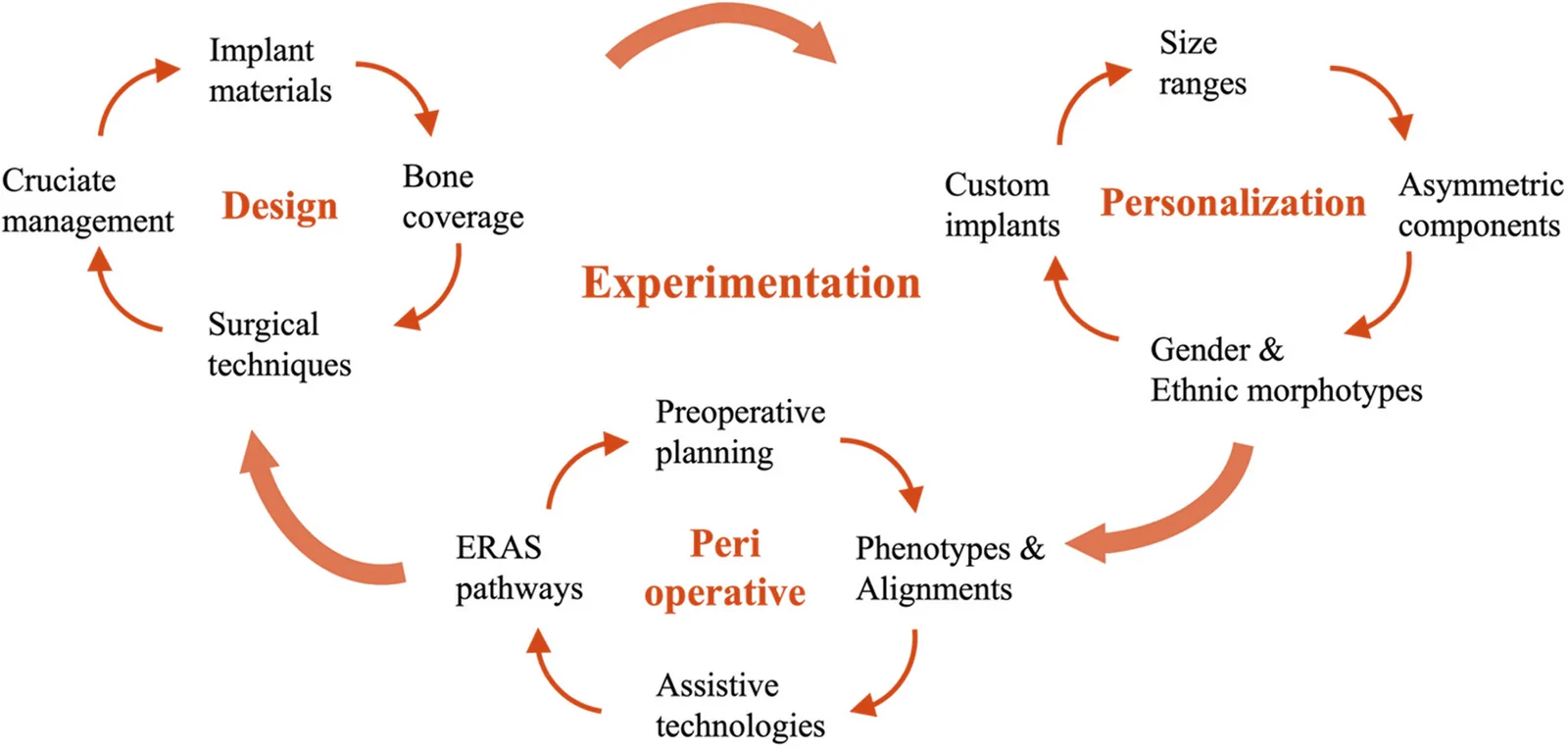
Evolutionary aspects of TKA

**Fig. 2:**
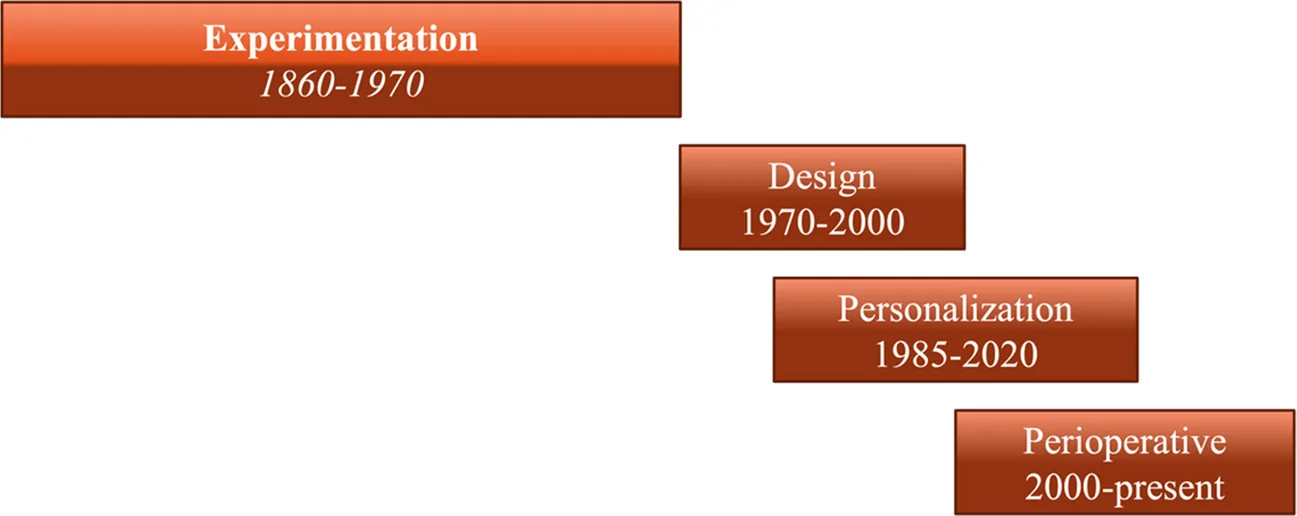
Evolutionary phases of TKA

The history of TKA may be divided into four major evolutionary phases (Figs. 1, 2): the era of experimentation between 1860 and 1970, the period of implant design and biomechanical standardization between 1970 and 2000, the rise of personalization and morphologic understanding between 1985 and 2020, and the modern era of perioperative and technological evolution beginning in the early 2000’s. Understanding this historical progression is important because many contemporary controversies, including alignment philosophy, implant personalization, robotic surgery, and ligament management, are direct extensions of earlier concepts.

## Phase I: Experimentation (1860–1970)

### Early soft-tissue interposition procedures

**Fig. 3:**
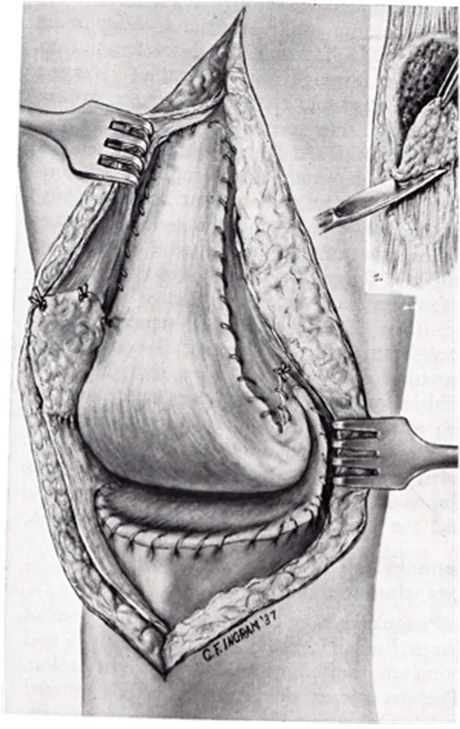
The knee arthroplasty as described by Campbell used the contralateral Fascia Lata. From: Waring TL Arthroplasty In Campbell’s operative orthopaedics. Crenshaw editor. The C.V. Mosby Company Saint Louis (MO) USA, 1963, p 1081

The earliest attempts at knee reconstruction emerged during the nineteenth century as surgeons sought alternatives to arthrodesis and amputation for tuberculosis, ankylosis, and degenerative arthritis. In 1861, William Fergusson described excision arthroplasty techniques intended to preserve knee motion after resection of diseased articular surfaces (Fig. 3) [2]. Shortly afterward, Peter Price further detailed conditions requiring knee excision and emphasized preservation of function whenever possible [3]. During the late nineteenth and early twentieth centuries, surgeons experimented with biologic interposition arthroplasty using fascia lata, muscle, fat tissue, pig bladder, nylon, and cellophane to reduce pain while maintaining mobility [4]. Although these procedures frequently resulted in instability, stiffness, and limited durability, they established the foundational principle of joint resurfacing rather than joint fusion.

### Metallic resurfacing and mold arthroplasty

**Fig. 4:**
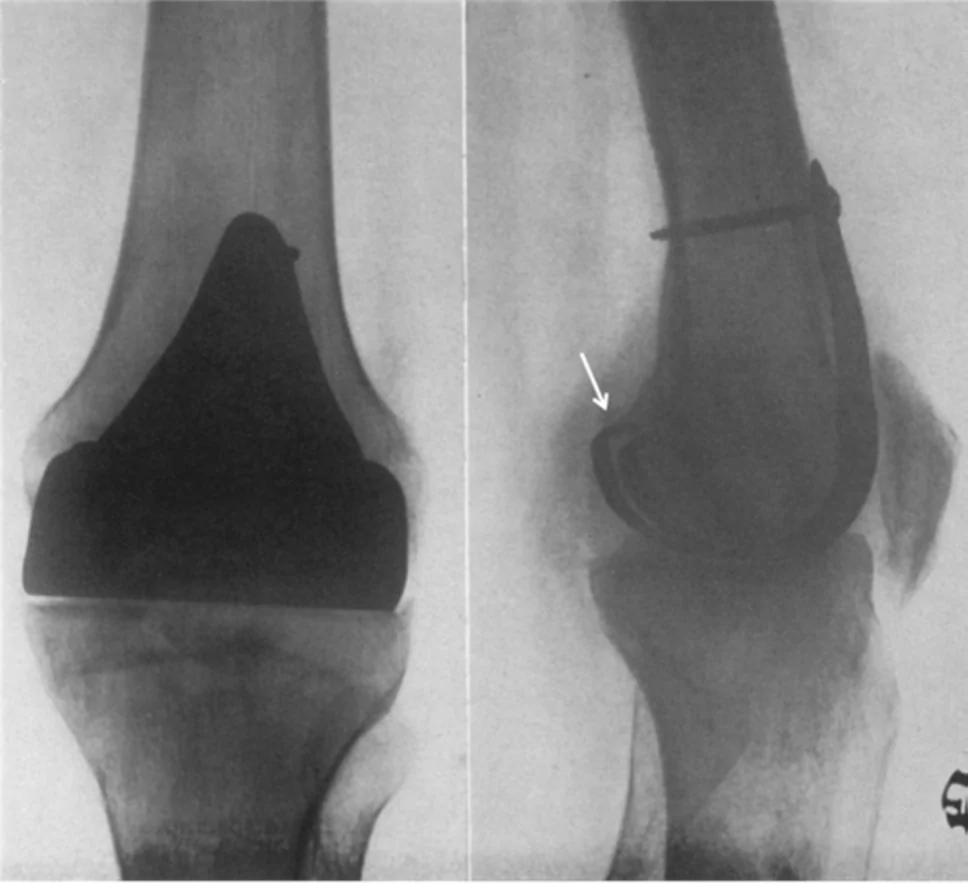
The vitallium knee arthroplasty of Campbell. From Campbell W (1940) interposition of vitallium plates in arthroplasties of the knee. Am J Surg 47 (3):639–641. With permission

By the 1920 s and 1930 s, arthroplasty concepts shifted progressively from biologic interposition toward prosthetic resurfacing. Campbell introduced molded vitallium femoral implants in 1940 to resurface the distal femur while preserving tibial bone stock (Fig. 4) [4]. These implants were custom-sized using radiographic templating and represented some of the earliest attempts at metallic femoral resurfacing. Jones later described mold arthroplasty techniques that further refined resurfacing procedures [5]. These early systems introduced concepts still central to modern arthroplasty, including individualized implant design, preservation of bone stock, restoration of articular geometry, and minimal bone resection.

### The first hinged prostheses

**Fig. 5:**
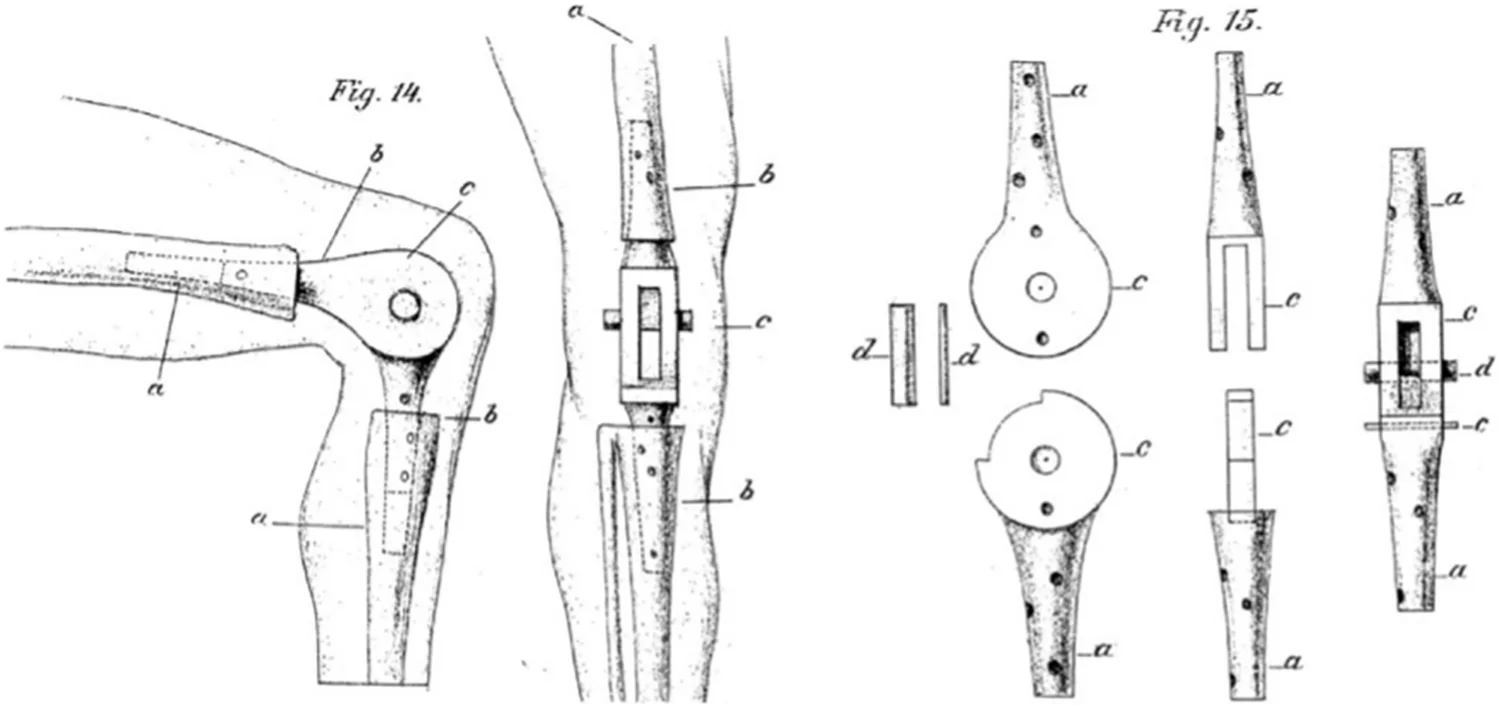
The first hinged prosthesis of Glück. From: Brand RA, Mont MA, Manring MM (2011) Biographical sketch: Themistocles Gluck (1853–1942). Clin Orthop Relat Res 469 (6):1525–1527. With permission

One of the earliest true total joint replacement concepts was introduced by Themistocles Gluck in 1890, when he implanted an ivory hinged knee prosthesis fixed with primitive cement materials (Fig. 5) [6]. Although infection and mechanical failure limited clinical success, Gluck established the conceptual principle of total joint replacement. His work coincided with important technological advances such as antiseptic surgery, radiography, anesthesia, and sterile surgical techniques, which collectively created the environment necessary for future arthroplasty development.

### Development of hinged knee arthroplasty

Modern hinged knee arthroplasty emerged during the 1950 s as surgeons attempted to address severe arthritis and ligamentous instability, which constrained out of plane movement by allowing only flexion–extension. Walldius initially introduced acrylic hinge prostheses before transitioning to cobalt-chromium implants with improved mechanical durability [7, 8]. These systems attempted to mechanically substitute for deficient ligament function using constrained tibial articulation. Wilson later reported clinical results using the Walldius prosthesis in rheumatoid arthritis patients [9]. Additional constrained systems, including the Guepar prosthesis [10], followed shortly thereafter. Although these implants provided pain relief and stability, excessive constraint generated high stresses at the bone-cement interface, resulting in loosening, infection, polyethylene wear, and mechanical failure.

### Emergence of unicompartmental arthroplasty

At the same time, minimally invasive arthroplasty concepts emerged. McKeever developed metallic tibial plateau hemiarthroplasty without major bone resection [11]. MacIntosh subsequently introduced unicompartmental prostheses composed of acrylic, Teflon, titanium, and vitallium [12]. These procedures preserved both cruciate ligaments and attempted to maintain native knee kinematics while treating isolated compartment disease. Although early survival rates was inconsistent, these systems established the conceptual foundation for modern unicompartmental knee arthroplasty.

### The polycentric knee and early condylar concepts

**Fig. 6:**
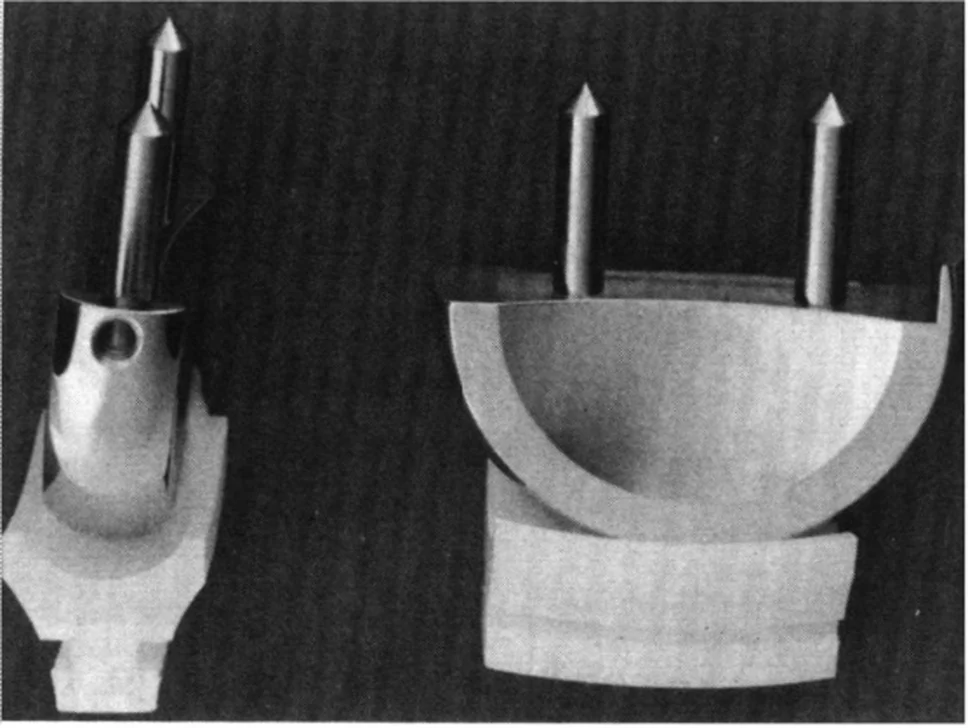
The Gunston prosthesis (Polycentric knee). From Ranawat Anil S, Ranawat Amar S, Ranawat Chitranjan S The history of Total Knee Arthroplasty. In Bonnin M, Amendola N, Bellemans J, MacDonald S and Ménétrey J. The Knee Joint: surgical technique and strategy. Springer. Paris. 2012. P697-709. With permission

**Fig. 7:**
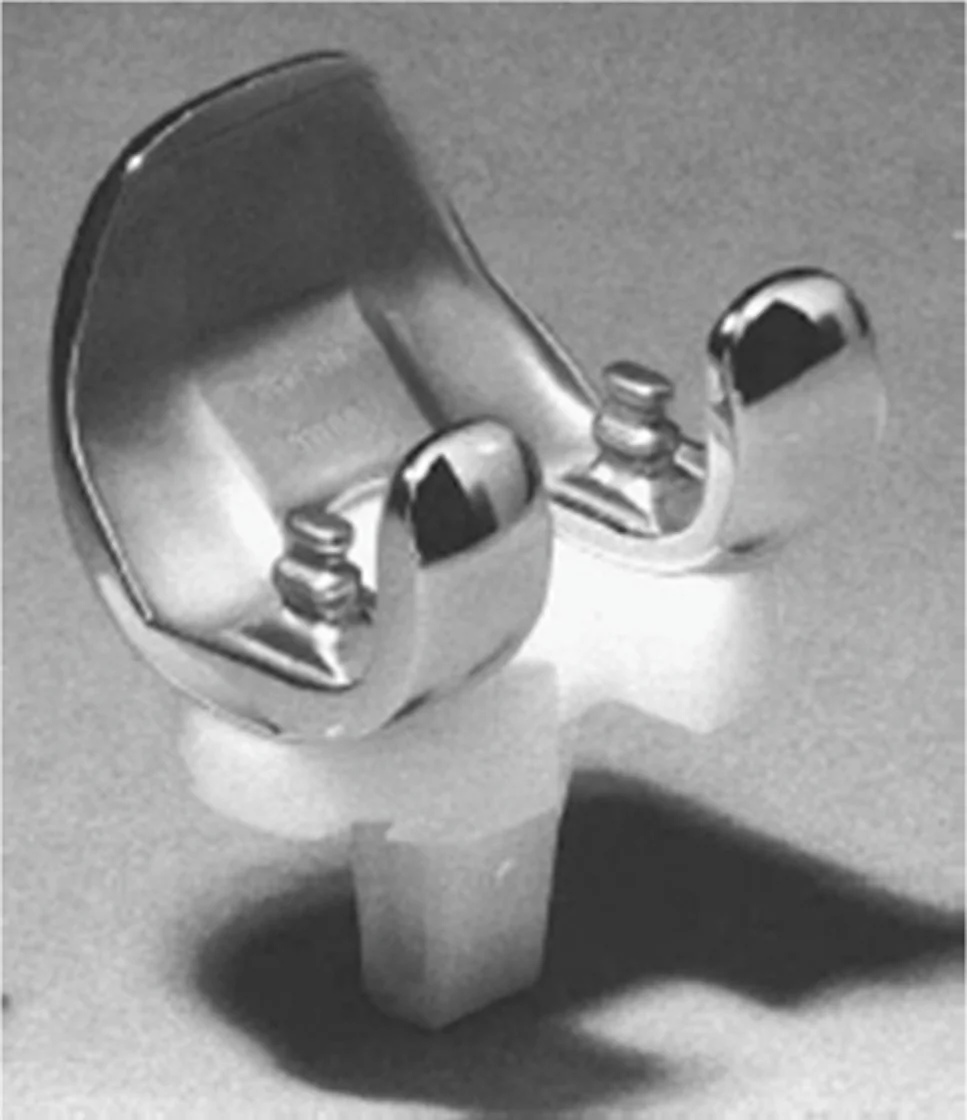
The Duopatella prosthesis. From Ranawat Anil S, Ranawat Amar S, Ranawat Chitranjan S The history of Total Knee Arthroplasty. In Bonnin M, Amendola N, Bellemans J, MacDonald S and Ménétrey J. The Knee Joint: surgical technique and strategy. Springer. Paris. 2012. P697-709. With permission

**Fig. 8:**
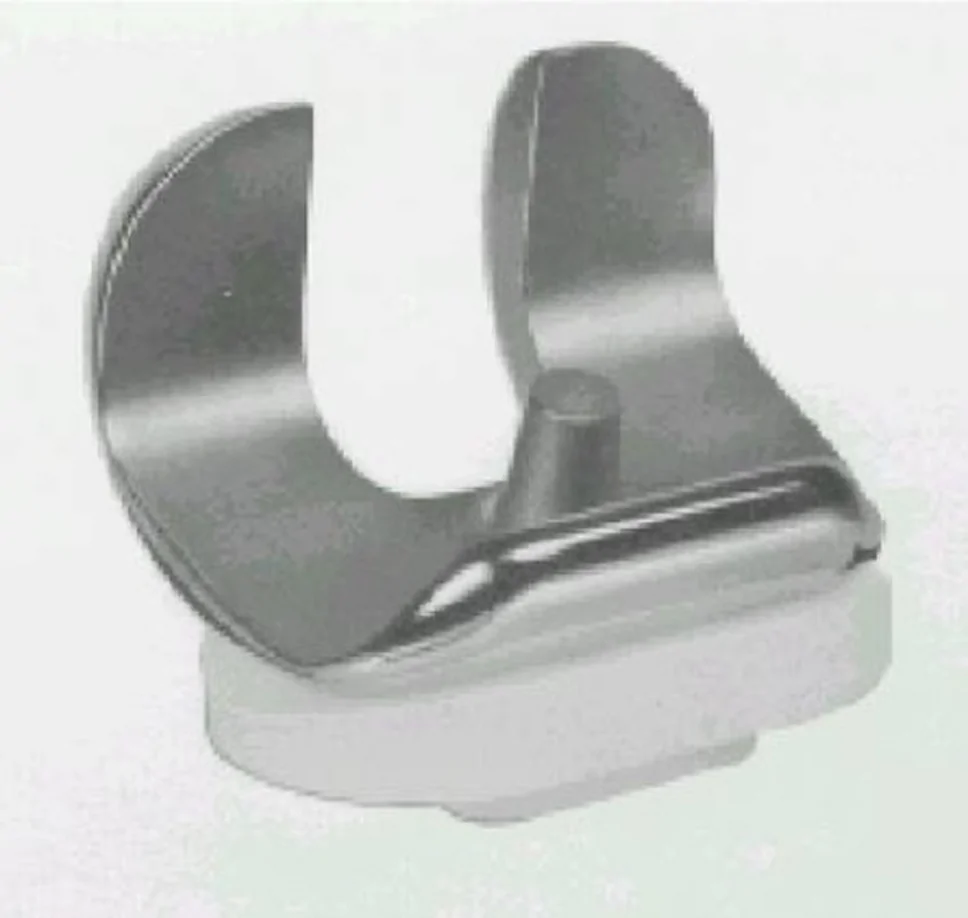
The ICLH prosthesis. From Ranawat Anil S, Ranawat Amar S, Ranawat Chitranjan S The history of Total Knee Arthroplasty. In Bonnin M, Amendola N, Bellemans J, MacDonald S and Ménétrey J. The Knee Joint: surgical technique and strategy. Springer. Paris. 2012. P697-709. With permission

By the late 1960 s and early 1970 s, surgeons increasingly recognized the biomechanical limitations of highly constrained hinged systems. In 1968, Frank Gunston introduced the polycentric knee (Fig. 6), inspired by low-friction arthroplasty principles and designed to reproduce more physiologic knee motion using separate tibiofemoral components [13]. However, excessive conformity between the articulating tibiofemoral surfaces resulted in high failure rates and mechanical complications [14]. Shortly thereafter, Ranawat and colleagues developed the Duocondylar and DuoPatella prostheses, representing a major transition toward modern condylar arthroplasty. The DuoPatella was among the first designs to replace the femoral trochlea, marking an important step toward comprehensive resurfacing in total knee arthroplasty (Fig. 7) [15]. Freeman and Swanson further advanced condylar principles through development of the Imperial College London Hospital (ICLH) prosthesis (Fig. 8) [16]. The ICLH philosophy emphasized careful ligament balancing and selective soft tissue release to achieve stability throughout the range of motion, an approach advocated by Dr. Van Vuuren that influenced modern TKA practice. These systems shifted TKA philosophy away from rigid hinge mechanics toward reproduction of native knee kinematics.

## Phase II: Implant design and biomechanical standardization (1970–2000)

### The total condylar prosthesis

In 1974, Insall and colleagues at the Hospital for Special Surgery introduced the Total Condylar prosthesis, widely considered the foundation of modern TKA [17]. This design established several principles that remain fundamental today, including measured resection techniques, surgical instrumentation, modular component positioning, improved patellofemoral mechanics, and balanced tibiofemoral conformity. The Total Condylar prosthesis demonstrated excellent mid-term survival rates and clinical outcomes, with 94% good or excellent results at five to nine years [18], and became the prototype for modern condylar arthroplasty.

### Posterior-stabilized designs

To compensate for posterior cruciate ligament sacrifice and improve flexion kinematics, Insall and Burstein introduced the posterior-stabilized post-cam mechanism in 1978 [19]. The post-cam design reproduced femoral rollback during flexion and improved stability throughout the range of motion. Posterior-stabilized systems rapidly became one of the dominant TKA philosophies worldwide and various subsequent versions are still being used today**.**

### Bi-cruciate and medial pivot concepts

**Fig. 9:**
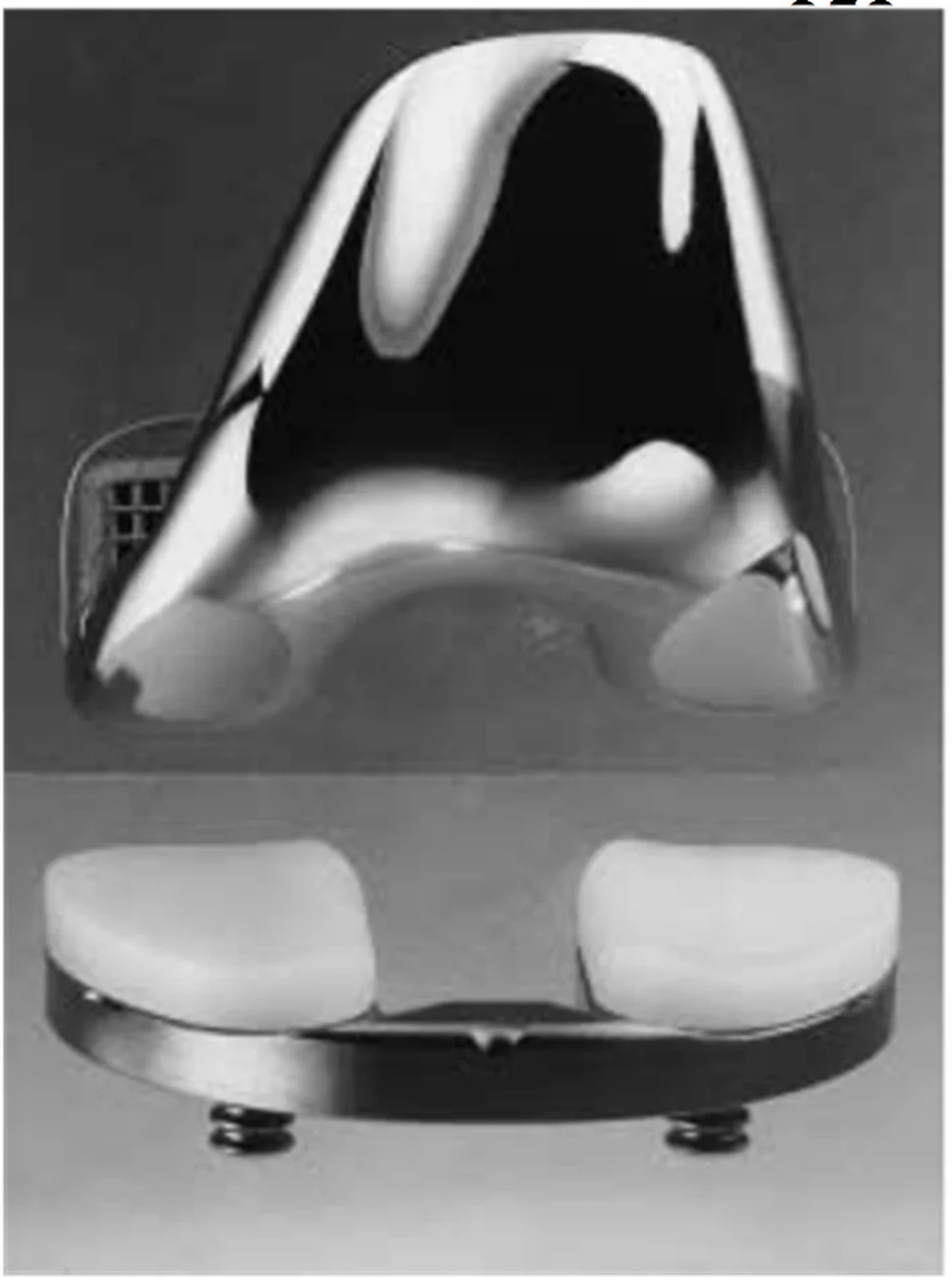
The Cloutier bicruciate-retaining knee prosthesis

**Fig. 10:**
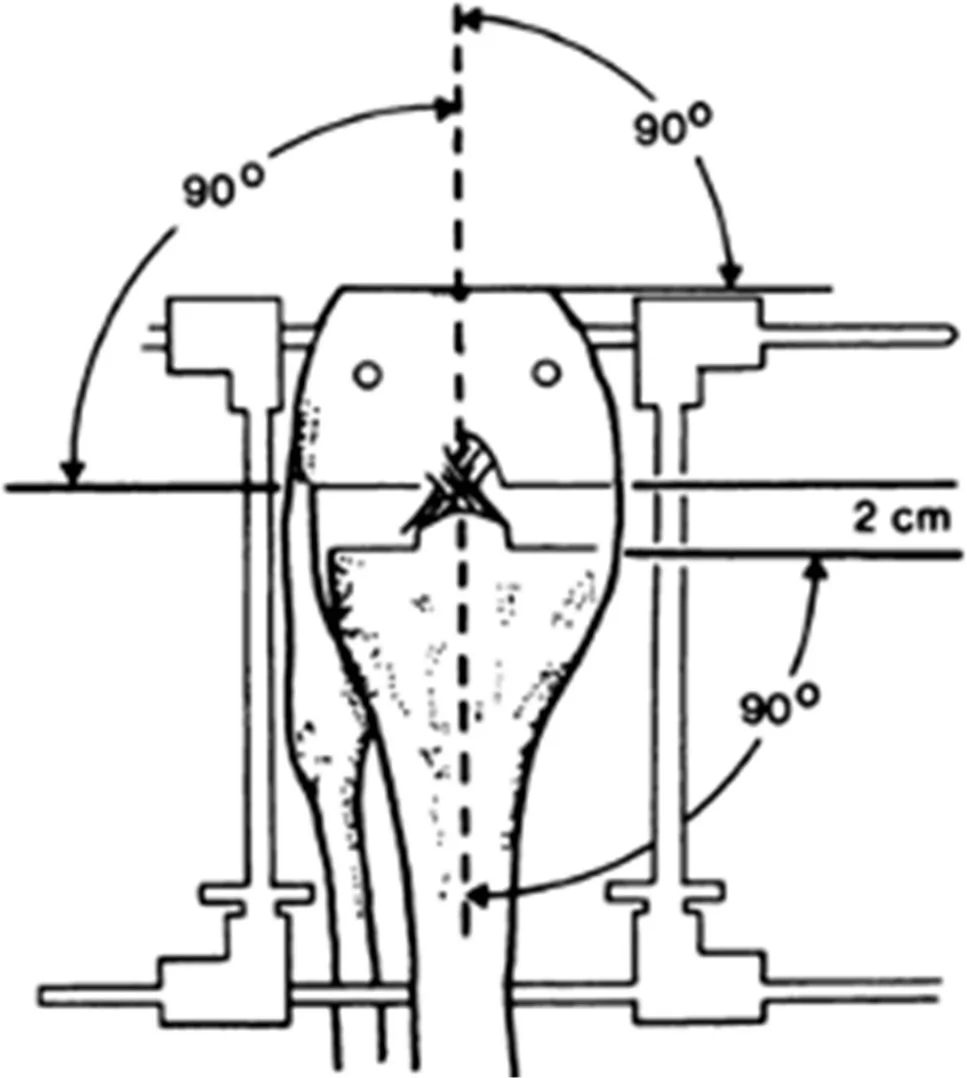
Schematic illustration of the Cloutier bicruciate-retaining total knee arthroplasty

During the late twentieth century, additional philosophies emerged in response to persistent limitations of conventional systems. Cloutier developed bicruciate-retaining arthroplasty concepts intended to preserve both cruciate ligaments and restore more native knee kinematics (Figs. 9, 10) [20]. Concurrently, increasing understanding of native knee biomechanics stimulated development of medial pivot systems designed to reproduce the screw-home mechanism and physiologic rotational behavior of the knee [21]. These concepts showcased that successful TKA required restoration of physiologic motion rather than simple mechanical alignment.

### Cemented and cementless fixation

Fixation philosophy evolved substantially during this era. Cemented fixation using polymethylmethacrylate became the gold standard because of reliable early stability and reproducible clinical outcomes. However, interest in biologic fixation emerged rapidly during the late 1970 s and early 1980s. Hungerford, Kenna, and Krackow introduced porous-coated cementless systems intended to promote osseointegration [22]. Although early cementless implants experienced variable fixation and polyethylene wear complications, these systems laid the groundwork for modern biologic fixation technologies.

### Modularity and mobile bearings

**Fig. 11:**
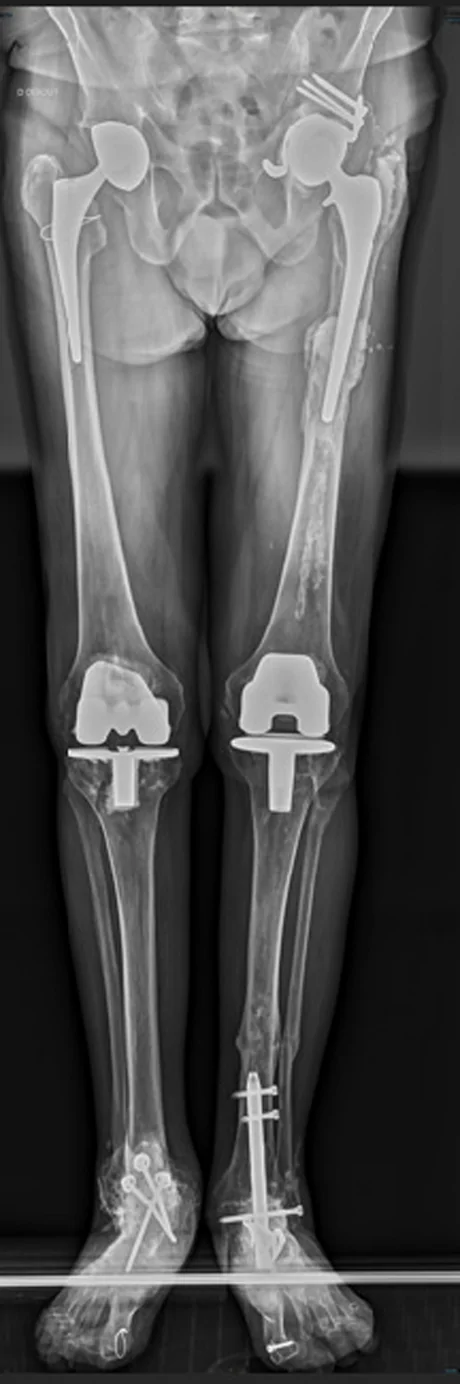
Standing anteroposterior radiograph showing the Insall-Burstein II (IB II) modular total knee arthroplasty 35 years after implantation (left knee)

During the 1980 s, modularity and mobile-bearing concepts gained widespread popularity. Metal-backed tibial components allowed polyethylene exchange and improved intraoperative flexibility, a concept exemplified by the Insall Burstein Posterior Stabilized II knee prosthesis, which introduced a modular tibial tray capable of accommodating augments and stem extensions (Fig. 11). Simultaneously, Goodfellow and O’Connor pioneered mobile-bearing concepts designed to reduce polyethylene wear through improved conformity while preserving mobility [23]. Buechel and Pappas subsequently introduced the Low Contact Stress rotating-platform knee, which further advanced mobile-bearing technology [24]. By the late 1980 s, the major foundations of modern TKA had been established, including modular implants, fixed and mobile bearings, cemented and cementless fixation, ligament balancing, and standardized instrumentation.

**Fig. 12:**
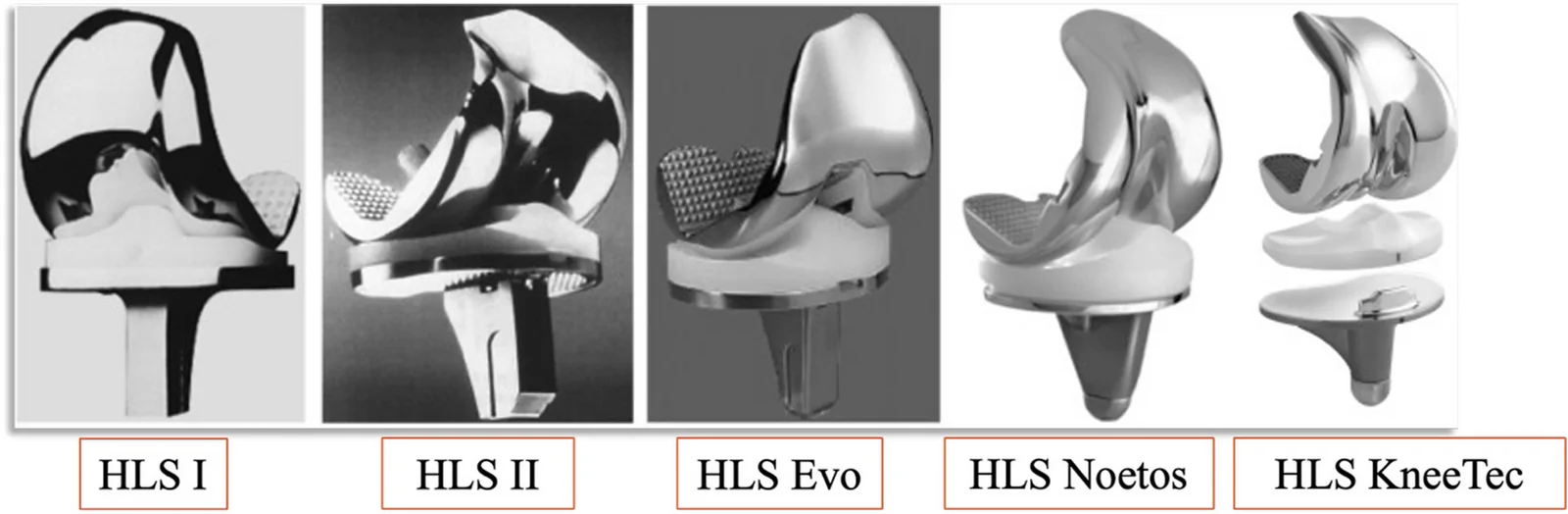
Evolution of 3rd condyle Hôpital Lyon Sud (HLS) system

French surgeons Dejour, Chambat, and Deschamps later developed the Hôpital Lyon Sud (HLS) system using a “third condyle” mechanism to reduce tibial loosening and improve physiologic rollback (Fig. 12) [25]. These developments reflected growing emphasis on reproducing native knee biomechanics rather than simple mechanical motion.

## Phase III: Personalization and morphologic understanding (1985–2020)

### Recognition of anatomic variability

Beginning in the late 1980 s and accelerating during the 1990 s and early 2000 s, researchers increasingly recognized that standardized implant geometries did not accurately reproduce native anatomy in many patients. Advanced CT- and MRI-based morphometric studies demonstrated substantial variability in distal femoral morphology, tibial anatomy, trochlear orientation, and sagittal curvature [26, 27]. Hitt and colleagues introduced the concept of implant “aspect ratio,” highlighting mismatch between standard implants and native knee anatomy [26]. Additional studies demonstrated important differences associated with sex, ethnicity, constitutional alignment, and body habitus [27, 28].

### Gender-specific and morphology-based designs

Recognition of morphologic variability stimulated development of expanded implant ranges and gender-specific prostheses during the early 2000s. Studies by Bellemans, Lonner, and others demonstrated substantial differences in mediolateral dimensions and distal femoral morphology between male and female knees [28, 29]. Barrett and colleagues advocated gender-specific implant systems intended to improve fit and reduce overhang [30]. Although the long-term superiority of gender-specific implants remains controversial, these developments represented an important transition toward personalized arthroplasty. Increasing evidence also demonstrated that implant overhang and under coverage could contribute to pain, stiffness, instability, and patellofemoral complications.

### Alignment philosophy evolution

During the 2000 s and 2010 s, alignment philosophy underwent a major paradigm shift. Traditional mechanical alignment sought universal neutral alignment, but studies demonstrated that many patients naturally possess constitutional varus or valgus alignment [31]. At the same time, Berger and colleagues emphasized the importance of rotational alignment and demonstrated complications associated with femoral malrotation [32]. In vivo kinematic studies further showed that native knee motion involved asymmetric rollback and dynamic ligament interaction [33, 34].

Consequently, personalized philosophies such as kinematic alignment, restricted kinematic alignment, inverse kinematic alignment, and functional alignment emerged. These approaches aimed to restore native joint-line orientation and physiologic soft-tissue balance rather than imposing standardized alignment targets [35].

### Custom and patient-specific implants

As appreciation of anatomic variability increased, efforts shifted toward developing implants tailored to individual patient morphology. Conventional off-the-shelf implants often failed to accurately reproduce native femoral and tibial anatomy, leading to concerns regarding component overhang, undercoverage, and altered knee kinematics. Advances in CT- and MRI-based imaging, computer-aided design, and manufacturing technologies enabled the development of patient-specific implants designed to improve implant fit and better restore native joint geometry [36]. Systems such as the ConforMIS iTotal and later custom implant platforms utilized preoperative three-dimensional imaging to create individualized femoral and tibial components based on each patient's anatomy.

## Phase IV: Technological and perioperative evolution (2000–Present)

### Computer navigation and digital planning

The twenty-first century introduced major advances in digital surgery. Computer-assisted navigation systems were developed to improve alignment precision and implant positioning by providing real-time intraoperative feedback regarding bone cuts, component orientation, and limb alignment [37]. Although navigation improved radiographic accuracy and reduced alignment outliers, long-term clinical superiority over conventional instrumentation remains inconsistent. Nevertheless, navigation laid the technological foundation for robotic-assisted arthroplasty.

### Patient-specific instrumentation

Patient-specific instrumentation (PSI) gained popularity during the late 2000 s as surgeons sought individualized surgical planning and simplified workflows. PSI systems used MRI or CT-based imaging to manufacture customized cutting guides specific to patient anatomy[38]. Although early enthusiasm focused on shorter operative times and improved alignment, later studies generally demonstrated outcomes comparable to conventional instrumentation [39]. PSI nevertheless represented an important intermediate step toward personalized digital arthroplasty.

### Robotic-assisted TKA

**Fig. 13:**
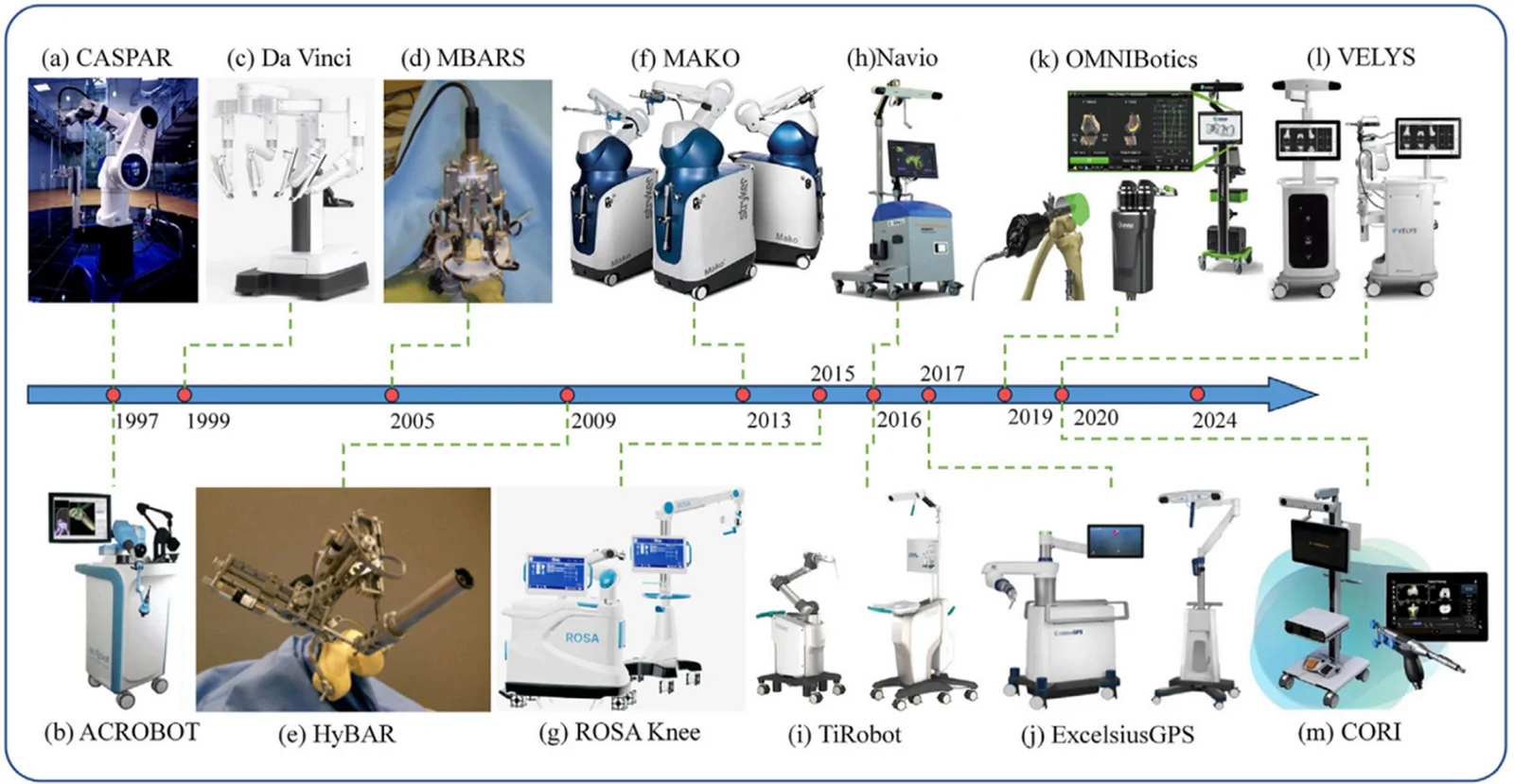
Physical drawings of various orthopedic robotic products, organized by product launch timeline. **A** CASPAR. **B** ACROBOT. **C** Da Vinci. **D** MBARS. **E** HyBAR. **F** MAKO. **G** ROSA Knee. **H** Navio. **I** TiRobot. **J** ExcelsiusGPS. **K** OMNIBotics. **L** VELYS. **M** CORI

Robotic-assisted TKA became one of the most important contemporary developments in knee arthroplasty during the 2010 s (Fig. 13). Modern systems such as MAKO, ROSA, CORI, and OMNIBotics integrate three-dimensional imaging, navigation, haptic guidance, and dynamic ligament balancing into a single surgical platform. Robotic systems may be classified as image-based, using preoperative CT imaging for three-dimensional planning, or imageless, relying on intraoperative anatomical mapping [40]. They are also categorized as autonomous, which perform bone preparation actively or semi-autonomous, which maintain surgeon control while restricting bone cuts within predefined boundaries, and are currently the most widely adopted robotic systems.

These technologies improve the precision and reproducibility of bone resections, reduce alignment outliers, facilitate personalized alignment strategies, and enhance intraoperative soft-tissue balancing. The OMNIBotics system uniquely incorporates a dynamic ligament tensioner that quantitatively evaluates medial and lateral soft-tissue balance before bone resection, allowing implant positioning to be adjusted according to ligament tension [40]. Although robotic-assisted TKA consistently improves alignment accuracy and component positioning, evidence for superior patient-reported outcomes, functional recovery, or implant survival rates remains inconsistent [41]. Consequently, the clinical utility and cost-effectiveness of robotic TKA remain debated, and it is still unclear which outcome measures best reflect the long-term value of robotic systems [42].

### Enhanced recovery after surgery (ERAS)

Modern TKA evolution extended beyond implant design and surgical technology into perioperative management. Enhanced Recovery After Surgery (ERAS) pathways introduced a structured, multidisciplinary approach aimed at reducing surgical stress, complications, and recovery time through evidence-based, patient-centered care [43]. Key components include multimodal analgesia, regional anesthesia, tranexamic acid administration, blood conservation strategies, early mobilization, and accelerated rehabilitation [44]. These protocols have significantly reduced hospital stay and postoperative morbidity while improving functional recovery and patient satisfaction following TKA [45].

### Current challenges and future directions

Despite the excellent survival rates of modern TKA, patient dissatisfaction remains an important challenge. DeFrance et al. [46] reported a dissatisfaction rate of approximately 10% following TKA, with the literature identifying persistent pain, stiffness, limited range of motion, complications, and unmet expectations as the main contributors to dissatisfaction.

The future of total knee arthroplasty is likely to be driven by the convergence of advances in implant design, surgical philosophy, digital technologies, and biologic reconstruction. Rather than representing isolated innovations, these developments constitute the natural continuation of the historical trend toward reproducing native knee anatomy, biomechanics, and function. Future implant designs will likely move beyond conventional off-the-shelf components toward increasingly individualized solutions. Improvements in three-dimensional imaging, additive manufacturing, and computational modeling may facilitate wider adoption of patient-specific implants with optimized articular geometry, individualized sagittal and coronal contours, and improved restoration of ligament isometry. Advances in biomaterials (including highly cross-linked and vitamin E-stabilized polyethylene, porous trabecular metals, bioactive coatings, and surface modifications promoting osseointegration) may further enhance implant longevity while reducing aseptic loosening and polyethylene wear. Modular and adaptable implant systems capable of accommodating patient-specific anatomy and ligament function may become increasingly important.

Surgical techniques are also expected to continue evolving toward greater personalization. Alignment strategies will likely become increasingly individualized, integrating constitutional alignment, dynamic soft-tissue balance, and patient-specific functional goals rather than relying on universal mechanical targets. Improved understanding of knee kinematics may refine indications for cruciate-retaining, medial or dual pivot, and customized constraint designs, allowing surgeons to better match implant selection to individual ligament competence and morphology.

Technological assistance will probably play an increasingly central role throughout the perioperative pathway. Robotic-assisted surgery is expected to evolve beyond precise bone preparation toward intelligent surgical platforms capable of integrating preoperative imaging, intraoperative soft-tissue assessment, predictive biomechanical modeling, and real-time decision support. Artificial intelligence and machine learning may facilitate automated surgical planning, prediction of patient-specific outcomes, optimization of implant positioning, and individualized rehabilitation protocols. The integration of augmented reality, computer vision, digital twins, and sensor-enabled "smart" implants capable of monitoring joint loading, alignment, and functional recovery may further improve postoperative surveillance and enable truly data-driven arthroplasty.

Ultimately, the future of TKA will likely extend beyond improving implant survival alone. The principal objective will be to restore a knee that behaves as closely as possible to the native joint while maximizing patient satisfaction, functional performance, and quality of life. As demonstrated throughout the history of TKA, each technological advance has progressively shifted the field from simply replacing the arthritic joint toward recreating its anatomy, biomechanics, and physiological function. The next generation of innovations is therefore expected to further personalize treatment, combining advanced implant engineering, precision surgery, and digital technologies to achieve truly individualized knee arthroplasty.

## Data Availability

No datasets were generated or analysed during the current study.
